# Contraceptive practices among women seeking termination of pregnancy in one public hospital in Eastern Cape, South Africa

**DOI:** 10.4102/phcfm.v8i1.1094

**Published:** 2016-08-31

**Authors:** Ebenezer O. Oluwole, Linda Skaal

**Affiliations:** 1 Department of Public Health, University of Limpopo, South Africa

## Abstract

**Background:**

There is significantly high contraceptive knowledge in South Africa, but the uptake of contraceptives is average to low with resultant soaring of unplanned pregnancy and rising statistics of termination of pregnancy (TOP) services. This study aimed to establish the contraceptive practices among women in the South African population seeking TOP in one public hospital in Eastern Cape, South Africa.

**Methods:**

A cross-sectional study was carried out among women seeking TOP in a women’s clinic. Self-administered questionnaires were used as data collection tool, and the data collected were entered into SPSS software for analysis, using descriptive statistics to calculate frequencies and percentages while chi-square test was used to determine the associations between the socio-demography and contraceptive practices of the participants.

**Results:**

Majority of the women were aged between 20 and 29 years, had secondary education, unemployed, single and resided in townships. Contraceptive uptake prior to termination of pregnancy (CTOP) among them was 44.1%, but 85.8% had good contraceptives knowledge. Their contraceptive practices are determined by partner’s opinion, source and availability of contraceptives, previous CTOP, side effect of contraceptives and having children. Age group, educational level and employment status were found to be related to the contraceptive practices of the participants but were not statistically significant.

**Conclusion:**

To reduce unplanned pregnancies and subsequent number of women seeking CTOP, the socio-economic factors associated with contraceptive practices as well as the programmes, policies and guidelines of contraceptives need to be improved on for any improvement on the factors determining contraceptive practices.

## Background of the study

The use of contraception to prevent pregnancy and its increased use among sexually active individuals can result in a significant reduction in unwanted pregnancies and resultant abortions. Globally, according to an estimation, 205 million pregnancies occur annually, with approximately 68 million of these pregnancies being unplanned pregnancies and 41 million of them ending in abortion.^1^ It is reported that over 200 million women prefer to delay their next pregnancy or not have children any more in developing countries, including South Africa (RSA); however, the majority of these women practice traditional and less-effective contraceptive methods or do not use any contraceptives.^1^

Many reasons account for poor uptake of contraceptives among women and they include lack of access, lack of awareness, cultural factors, religion, opposition to use by partners or family members, and fear of health risks and side effects of contraceptives.^3^ It is estimated that 90% of the abortion-related and 20% of the pregnancy-related morbidity and mortality can be prevented with the use of effective contraception.^4^ It is further reported that unintended pregnancy poses a major problem to reproductive health delivery in developing countries such as South Africa. Studies report that 35% of women become pregnant before the age of 20 years in South Africa and this has a significant impact because of economic, social, psychological and physical implications for those with unplanned pregnancies.^5^

Knowing the contraceptive practices of these women in the study will serve as pointer to where the service providers are failing and need refocusing and re-strategising.^6^

According to the findings of World Health Organization and Guttmacher Institute,^6^ it is reported that in low-income countries, where fertility rate is quite high, non-contraceptives usage contribute to two-thirds of unintended pregnancies and 11% of these lead to unsafe abortion which result in severe complications such as infections and bleeding.^7^ These unwanted pregnancies can be avoided by mere use of contraceptives.^7^

The South African Saving Mothers Report^8^ reports that maternal mortality rate increased from 152/100 000 live births in 2005–2007 to 176/100 000 live births in 2008–2010 in South Africa. Magadi and Curtis,^9^ found that wider range of contraceptive methods is directly proportional to higher contraceptive demand. Availability of a wider range of contraceptives has been found to give the women options to choose what is best for their contraceptive needs and thereby reduce chances of unplanned pregnancies that often result in termination of pregnancy (TOP).^10^

The use of contraceptives for prevention of unwanted pregnancy by women is one of the United Nations (UN) organisation’s strategies in achieving Millennium Development Goal to improve maternal health.^4^ However, prevention of unwanted pregnancy requires quality reproductive health services so that women can access safe and efficient methods of contraception.^4^

The South African government has put in place various programmes, strategies and policies to facilitate the use of contraceptive services as a step to reducing fertility rates and increasing contraceptives uptake and therefore reducing poor maternal outcomes. However, a number of women seeking TOP at public hospitals in South Africa are rising. Therefore, understanding the characteristics of the women in this population, their contraceptive practices and the factors influencing contraceptive use may provide possible areas/factors that could be targeted for interventions to prevent unplanned pregnancies, leading to subsequent reduction in termination of pregnancies. However, a number of women seeking TOP at public hospitals, such as in South Africa, are rising.^11^ The aim of this study was to establish the contraceptive practices among women seeking TOP in Dora Nginza Hospital, Port Elizabeth, Eastern Cape, South Africa.

## Research methodology

### Study design and approach

The researchers used a descriptive cross-sectional design, quantitative study approach, targeting women aged 12–49 who were seeking TOP at the public hospital. Permission to conduct the study and ethical clearance were obtained from the Department of Health and Medunsa Research Ethics Committee, respectively.

### Sampling and sampling techniques

Consecutive women attending the TOP clinic were approached to participate in the study and those who agreed to participate and met the inclusion criteria were included. A sample size of 339 was required based on an expected frequency of 50% with 95% confidence intervals having a width of 5%. In order to compensate for possible attrition, a sample of 400 was intended. The clinic usually saw 15 patients a day for TOP and a total of around 2880 a year.

### Data collection tools

Structured researcher-administered questionnaire, consisting of 29 questions, was used to collect data. The questionnaires were in three languages – English, Afrikaans and isiXhosa based on the languages of the target population. The questionnaire contained various questions relating to the demographic profile of the participants, contraceptive practices and factors influencing contraceptive practices.

A pretest was conducted through piloting to address reliability, and content validity was ensured by the use of experts in the clinic and the supervisor during the development of the questionnaire.

### Recruitment

The researchers went to the women’s clinic every morning, introduced the research and explained the purpose of the study to the potential participants in the presence of the two registered midwives who were trained on data collection. The prospective participants at that point in time were addressed in clear terms of potential benefits, risks, inconvenience and obligations associated with the research that might reasonably be expected to influence their willingness to participate. This was done at the waiting area of the clinic before their primary consultation with the midwives.

Those women who opted to participate were taken to another room and informed consent forms were given to them; after which they completed the questionnaire.

### Data analysis

The data collected were entered into a Microsoft Excel spreadsheet, where it was edited and then imported into SPSS and EPI-INFO software for cleaning, management and analysis. Frequency distribution and descriptive statistics were used to summarise the socio-demographic variables, contraceptive practices and factors influencing contraceptive use which were expressed as means, standard deviation as well as median. Some of these data were also categorised, and percentages and proportions were calculated. Possible associations between independent variables such as the socio-demographic profile and the dependent variables such as the contraceptive practices as well as factors influencing the contraceptive practices of the women were determined using inferential analysis like cross-tabulation and chi-square test. Logic regression was utilised in determining the confidence intervals at 95% level of significance in order to predict feasible association among the variable at 0.05 level of significance and *p*-values less than 0.05 considered as statistically significant.

## Results

The results show that out of the 395 respondents, over half (55.4%) were aged between 20 and 29 years, about one-third were 30–39 years and 15.7% were less than 20 years of age. The majority (84.6%) of the participants were black people, 14.7% were from mixed race people and less than 1% were white people. The majority of the respondents were single (93.9%) and 72.7% were unemployed. About two-thirds (59.7%) of all the participants have secondary education status, 32.2% had tertiary education and just less than 10% had primary education or less. Also, over two-thirds (76.5%) of the participants reside in the township, 16.7% in the informal settlements and less than 7% in suburbs. Two-thirds (65.1%) were unemployed and only one-third (34.9%) were employed (Table 1).

**TABLE 1:** Demographic profile of the participants.

Variables	(*n* = 395)	Frequencies (*n*)	Percentages (%)
Age group	< 20 years	62	15.7
	20–29 years	219	55.4
	30–39 years	114	28.9
Race	Black people	334	84.6
	Mixed race people	58	14.7
	White people	3	0.8
Educational status	Primary or less	32	8.1
	Secondary	236	59.7
	Tertiary	127	32.2
Religion	Christians	378	95.7
	Muslim/Hindu	6	1.5
	Others	11	2.8
Marital status	Single	371	93.9
	Married	24	6.1
Residence	Township	302	76.5
	Informal settlement	66	16.7
	Suburb	27	7.8
Employment status	Employed	-	34.9
	Unemployed	-	65.1

*Source*: Authors’ own work

The results show the majority of the respondents (85.8%) reported that they knew about contraceptives, and 44.1% reported that they used contraceptives before pregnancy among the participants. One-third of the respondents reported that they heard about contraception from family (30.4%); 25.8% heard from healthcare workers and only 3.3% did not know about contraceptives. Furthermore, 33.9% of the respondents reported that they used condoms, 22.0% used injectable contraceptives and 32.7% (*n* = 129) did not use any contraception before pregnancy. Eighty-seven percent (87.4%) of the participants had no previous TOP while others had one or more. The results also show that a quarter (25.6%) of the respondents believe TOP is a form of contraceptive (see Table 2).

**TABLE 2:** Contraceptive practices of the participants.

Variables	(*n* = 395)	Frequency	Percentages (%)
Contraceptive knowledge	Yes	339	85.8
	No	56	14.2
Knowledge of hospital as source of contraceptives	Yes	297	75.2
	No	98	24.8
Use of contraception before pregnancy	Yes	174	44.1
	No	221	55.9
Source of information about contraceptives	Family	120	30.4
	Friends	69	17.5
	Healthcare workers	102	25.8
	Media	81	20.5
	Partner	10	2.5
	Not sure	13	3.3
Types of contraceptives used before pregnancy	None	128	32.6
	Injectable	87	22.0
	Oral	34	8.6
	Condoms	134	33.9
	Mixed	11	2.8
Previous TOP	None	345	87.4
	1	38	9.6
	2	10	2.5
	3	2	0.5
Use TOP as contraceptive	Yes	101	25.6
	No	295	74.4

*Source*: Authors’ own work

The results further show that one-third of the women were aware of contraceptive problems; 42.5% were aware of contraceptive advantages/benefits. From the results, already having children was perceived to be the determinant of contraceptive use by two-thirds of the participants, while number of children, contraceptive side effects, availability and source of contraceptives were perceived to determine use (42.8%; 43.8; 39.5%; 49.4%, respectively). The most commonly reported factor influencing contraceptive practices is the source of contraceptive (49.4%) and least reported factor is judgement by others (22.0%). Also, 31.4% of the respondents reported that partner’s opinion about contraceptive use affected contraceptive practices. Also, 40% of the participants reported that previous TOP determined contraceptive use and a quarter (25.6%) viewed TOP as a form of contraceptive. From the results, staff attitude (35.9%), partner’s opinion (31.4%), judgement by others (22.0%) and religious belief (26.3%) were perceived to determine contraceptive use by participants (see Table 3).

**TABLE 3:** Self-reported factors influencing contraceptives practices.

Factors	Yes *N* (%)	No *N* (%)
**Awareness of contraceptives**		
Awareness of contraceptive problems	137 (34.7)	258 (65.3)
Awareness of contraceptives advantages	168 (42.5)	227 (57.5)
**Determinants of contraceptive use**		
Already having other children	265 (67.1)	130 (32.9)
Number of children determine contraceptive use	169 (42.8)	226 (57.2)
Side effect of contraceptive influence use	173 (43.8)	222 (56.2)
Contraceptive availability determine use	156 (39.5)	239 (60.5)
Source of contraceptive determine use	195 (49.4)	200 (50.6)
**History of TOP**		
Previous TOP determine contraceptive use	158 (40.0)	237 (60.0)
TOP as form of contraceptive	101 (25.6)	294 (74.4)
**Perceived attitude**		
Staff attitude determine contraceptive use	142 (35.9)	253 (64.1)
Partner opinion determine contraceptive use	124 (31.4)	271 (68.6)
Judgement by others determine contraceptive use	87 (22.0)	308 (78.0)
Religious believe determine contraceptive use	104 (26.3)	291 (73.7)

*Source*: Authors’ own work

TOP, termination of pregnancy.

The results also show that two-thirds of the women at < 20 years, those at 29–30 years and 30–39 years reported that they were not using contraceptives prior to getting pregnant (66.1%, 53% and 56.1%, respectively) though not statistically significant (χ^2^ = 3.398; *p* = 0.183) (see Table 4).

**TABLE 4:** Cross-tabulation between age group and pre-pregnancy contraceptive use.

Variables		Pre-pregnancy contraceptive use		*P*†

		Yes *N* (%)	No *N* (%)	
Age group	< 20 years (*N* = 62)	21 (33.9)	41 (66.1)	
	20–29 years (*N* = 219)	103 (47.0)	116 (53.0)	0.183
	30–39 years (*N* = 114)	50 (43.9)	64 (56.1)	

*Source:* Authors’ own work

† , Chi-square: χ = 3.398.

## Discussion

The results of this study revealed that there is still an alarming number of black women seeking TOP in Eastern Cape, South Africa. Of the population size of 1880 women seeking TOP, a purposive sampling was used to select 395 women, majority of whom were black people. Several studies have reported similar race distribution when it comes to TOP, where black people remained the majority race seeking TOP compared to other races in most parts of South Africa.^10,12,13^ Of interest, this study found that most women between 20 and 29 years requested TOP more than any other age groups. At this age, it is assumed that women understand the risks of engaging in unprotected sexual activity or without contraceptive use, compared with adolescents. Of concern is that this is the era where HIV rates are escalating in South Africa; therefore, we need to focus on this group of young adults to raise awareness of the dangers of such behaviours. Currently, statistics report that more than 30% of women between 20 and 34 years were infected with HIV in this country^14^; it is therefore of concern that these women continue to engage in risky sexual behaviours, despite the fact that they are at high risk of contracting HIV.

Furthermore, this study found that only a quarter of the women seeking TOP were using contraceptives before they got pregnant. Contraceptive uptake is reported to be at 65% nationally, according to the South Africa Demographic Health Survey.^15^ Even in this study, contraceptive use prior to pregnancy was found to be low. Of concern is that some of women reported that they used condoms prior to pregnancy, however, incorrect and inconsistent use of any form of contraceptives have resulted in unplanned/unwanted pregnancies that led to these women seeking TOP in our study. Interestingly, we found that over half of the women reported that they were not using contraception before pregnancy, this, despite the fact that the majority of these participants had excellent-to-good contraceptive knowledge. Women with good knowledge about contraceptive use are expected to be more assertive and grounded in knowing which risks exist when one engages in unprotected sexual activity such as unwanted pregnancy, which could have been prevented by mere use of contraceptives that are free of charge in South Africa.

For those women who reported that they were using contraceptives prior to pregnancy, we found that they used a wide range of sources to seek contraceptive information such as families and healthcare workers, while a few relied on media and friends. These results show that families are beginning to show more interest regarding reproductive health than previously reported by Aderibigbe and Basebang.^15^ Also, unlike in other studies^16,17,18^ where media was found to be the major source of information on contraceptives, this study found only 20% of the respondents reported media to be their source of information on contraception. The disadvantages of media as a source of contraceptive information is that information could be easily misinterpreted, as it is brief and does not allow audience to seek clarity, contributing to risk of poor understanding of contraceptive use and resultant unwanted pregnancies if information is misinterpreted.

The significantly lower usage of contraception and comparably high contraceptive knowledge by the participants in this study is a concern because it shows that knowledge does not always translate to behaviour change in this group of women. This has also been found by several studies across Africa^19,20,21,22^ that reported that the low contraceptive use is directly related to poverty and illiteracy. In addition, up to half of women reported that availability and source of contraceptives were the determinants influencing the use of contraceptives. There is a necessity to promote contraceptive usage among these women within community settings in order to secure a much higher contraceptives uptake as recommended by the National Contraception Guidelines. This further indicates the need for continuous creation of awareness through various means about the available contraceptive services at the other sources. Additionally, the campaigns to be done should include increased outlets where contraceptives can be accessed, such as reinforcing accessibility within community settings.

Another significant finding in this study is that a quarter of the women viewed TOP as a form of contraceptive, implying that they are likely to terminate pregnancy in future. Already in this study, about 3% of women reported that they had had more than one TOP, all of which viewed TOP as a form of contraception, as found in similar studies in South Africa.^23,24,25^

## Limitations

The participants were chosen from the women seeking termination of pregnancies at the women’s clinic of Dora Nginza Hospital at the time of the study. Probably including women coming for other services at the hospital will give more information about availability, sources, access and other factors influencing the contraceptives practices.

In addition, the research was conducted in a health institution thereby revealing details of only the women seeking such services and may be different from the overall community. However, the study showed the participants were from widespread areas of the community including informal settlement, township and suburban areas.

Furthermore, the study was cross-sectional and therefore only revealed the contraceptive practices of the participants prior to pregnancy and not the changes over a period of time. A prospective study over a period of time is recommended to provide pattern of contraceptive practice over time in the community.

### Strengths

The most important strength of this study is the fact that it highlights the contraceptive practices of women who seek TOP in public hospitals. This information can be used to design intervention programmes specifically for women who seek TOP, in order for them to adopt a new behaviour of preventing unwanted pregnancy. Therefore, it adds to the existing body of knowledge and helps to fill the literature on contraceptive practices among women seeking TOP in South Africa.

### Recommendations

We feel that there is a need to include men in contraceptive programmes in order to improve joint family/partner collective decision-making when it comes to contraceptive use. We suggest that government must increase awareness in order to improve and encourage more contraceptives uptake among the women.

## Conclusion

This study provided us with relevant details on the contraceptive practices of women in terms of their socio-demographics, various practices involved with, as well as associations between their socio-demographic data and contraceptive practices. The study revealed that the contraceptive uptake was low prior to TOP, despite having a very high level of contraceptive knowledge and high level of education among the women seeking TOP in Port Elizabeth, South Africa. Knowledge of contraceptives in terms of side effects, sources and availability significantly determine contraceptive practices among the women seeking TOP in a hospital. Lastly, from the study it can be suggested that partner’s/spouse’s opinion is likely to play a significant role in the women’ decision on contraceptive usage, if incorporated in decision-making process of which contraceptive to use.
